# Malaria has no effect on birth weight in Rwanda

**DOI:** 10.1186/1475-2875-8-194

**Published:** 2009-08-10

**Authors:** Stephen Rulisa, Pètra F Mens, Corine Karema, Henk DFH Schallig, Nadine Kaligirwa, Joseph Vyankandondera, Peter J de Vries

**Affiliations:** 1 National University of Rwanda, Kigali University Teaching Hospital, BP 655, Kigali, Rwanda; 2 Center for Infection and Immunity Amsterdam, Center for Poverty Related Communicable Diseases (CPCD), Academic Medical Center, Meibergdreef 9, 1105 AZ Amsterdam, the Netherlands; 3 Royal Tropical Institute/Koninklijk Instituut voor de Tropen (KIT), KIT Biomedical Research, Meibergdreef 39, 1105 AZ Amsterdam, the Netherlands; 4 Center for Treatment and Research on AIDS, Malaria and TB (TRAC-PLUS) BP 2717, Kigali Rwanda; 5 Academic Medical Center, Division of Infectious Diseases, Tropical Medicine and AIDS, Meibergdreef 39, 1105 AZ Amsterdam, the Netherlands

## Abstract

**Background:**

Malaria has a negative effect on pregnancy outcome, causing low birth weight, premature birth and stillbirths, particularly in areas with high malaria transmission. In Rwanda, malaria transmission intensity ranges from high to nil, probably associated with variable altitudes. Overall, the incidence decreased over the last six years (2002–2007). Therefore, the impact of malaria on birth outcomes is also expected to vary over time and space.

**Methods:**

Obstetric indicators (birth weight and pregnancy outcome) and malaria incidence were compared and analyzed to their association over time (2002–2007) and space. Birth data from 12,526 deliveries were collected from maternity registers of 11 different primary health centers located in different malaria endemic areas. Malaria data for the same communities were collected from the National Malaria Control Programme. Associations were sought with mixed effects models and logistic regression.

**Results:**

In all health centres, a significant increase of birth weight over the years was observed (p < 0.001) with a significant seasonal fluctuation. Malaria incidence had no significant effect on birth weight. There was a slight but significant decreasing effect of malaria incidence on the occurrence of premature delivery (p-value 0.045) and still birth (p-value 0.009). Altitude showed a slight but significant negative correlation with birth weight. Overall, a decrease over the years of premature delivery (p = 0.010) and still birth (p = 0.036) was observed.

**Conclusion:**

In Rwanda, birth weight and pregnancy outcome are not directly influenced by malaria, which is in contrast to many other studied areas. Although malaria incidence overall has declined and mean birth weight increased over the studied period, no direct association was found between the two. Socio-economic factors and improved nutrition could be responsible for birth weight changes in recent years.

## Background

In sub-Saharan Africa, over 50 million women living in malaria endemic areas become pregnant each year [1] and *Plasmodium falciparum *malaria is an important contributor to low birth weight, maternal morbidity, preterm delivery, perinatal morbidity and mortality (still births) [2]. In areas with stable malaria transmission, *P. falciparum *infection during pregnancy is estimated to cause as many as 10,000 maternal deaths each year, contributing to approximately 2 – 15% of maternal anaemia, 8 – 14% of infants with LBW (an important contributor to infant mortality), and 3 – 8% of all infant deaths [3,4]. Although malaria in pregnancy is often asymptomatic, it nevertheless causes unfavourable pregnancy outcomes for both mother and child [5-7]. The invasion of the placenta by *Plasmodium *and the consequent inflammatory response is the cause for this pathology. Primigravid and secundigravid women have the highest risk of placental parasitaemia and malaria-associated LBW [6,8-10]. It is estimated that each year 75,000 – 200,000 infant deaths are associated with malaria in pregnancy [3,4]. Independent from malaria, LBW is the greatest risk factor for neonatal mortality and is a major contributor to infant mortality [11,12].

Most of the presented studies that associate maternal malaria and LBW use data from studies that compare individual patients in high transmission zones, where chronic parasitaemia is prevalent. Studies in low transmission areas are scarce as is information of the effect of low birth weight in Rwanda. Rwanda changed from a country with high placental malaria burden with 75% of the studied placenta's harboring parasites in 1998 [13] to an average of 13.6% placental malaria in 2005 [14]. The definition of malaria endemicity in Rwanda is difficult by the fact that transmission intensity varies with the large variation in altitude and is not continuously monitored. Based on a national malaria map by the National Malaria Control Programme/Programme National de Lutte contre le Paludisme (PNLP) of Rwanda from 1982, which is still used to date for classification of the country, the country is classified into four transmissions zones: 1) the high savanna area in the east of the country at 1,250 m to 1,500 m above sea level with a *Plasmodium falciparum *prevalence up to 80%; 2) 780 to 1,000 m above sea level with a *P. falciparum *prevalence varying between 30% to 50%; 3) the central plateau, situated at an altitude of 1,500 m to 1,800 m above sea level with a *P. falciparum *prevalence of 5% to 15%; 4) The Rusizi – Tanganyika plateau at an altitude of 1,800 to 3,000 m above sea level with a *P. falciparum *prevalence of 0 to 2%. It gives an indication of the differences between different regions but lacks details and does not take into account the variation in altitude within the transmission zones. Incidence data are widely available by the detailed reporting system of the curative health services. The PNLP and others [15] report that the malaria incidence in the country is decreasing over the last few years. This may also influence the causal effect of malaria on general health and in pregnancy in Rwanda, and this may not necessarily correspond to the description that is given to different transmission zones. In the present study, obstetric indicators (i.e. birth weight and outcome of pregnancy: at term, premature birth or still birth) and indicators of malaria incidence from local health registers of 11 different communities representing the different transmission zones were collected. The data were analysed to association between the outcome of pregnancy and malaria incidence while accounting for the variability and the decrease of malaria incidence in Rwanda.

## Methods

This was a retrospective, observational study comparing obstetric data from all pregnancy outcomes recorded from the 1^st ^to the 15^th ^of each month from January 2002 until December 2007 in different malaria endemic regions in Rwanda. Two types of existing data sources were used: birth registries of primary health centers (PHC) and malaria data sources from the PNLP. Ethical approval for this study was sought and obtained from the Rwanda National Ethics Committee (IRB1497)

### Study sites

**Figure 1:**
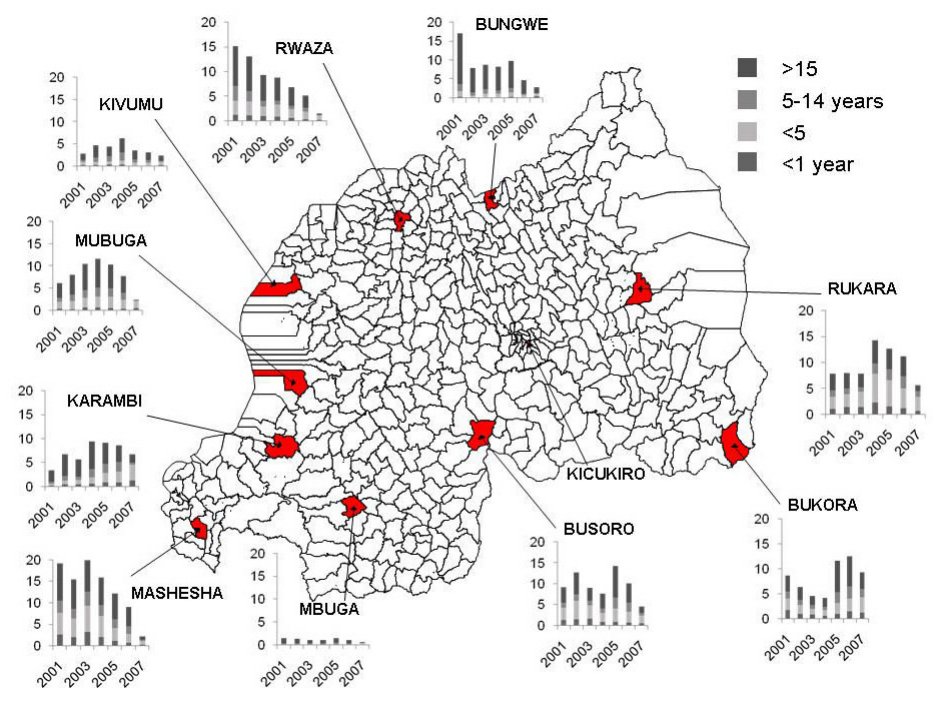
**Map of Rwanda showing the 11 studied communities marked in red**. Data on total malaria incidence (/1000 cap.) from 2001 – 2007 is presented in the column charts for each community, by age group and per year. Note: there are no data presented for Kicukiro in Kigali, because this site is not part of the National Malaria Control Programme sentinel sites.

Data were collected from 11 PHCs in Rwanda that represent the four different transmission zones in the Rwanda (Figure 1). The PHCs were selected on the basis of their participation as sentinel sites in a malaria surveillance programme of PNLP. The PNLP surveys the malaria incidence in sentinel sites, including the sites selected for this study, distributed over the country from regions with different transmission intensity. In order to include data on urban malaria, the PHC of Kicukiro in Kigali, which is not a PNLP sentinel site, was included in the present study. In the selected health centers diagnosis and treatment of malaria are subsidized and quality of diagnosis is continuously monitored.

### Malaria data

As an indicator for malaria transmission the malaria incidence data from the PNLP registers, corresponding to the period observed in this study, were used. The diagnosis of malaria at the sentinel PHCs was specified as confirmed malaria (microscopy positive) or as presumed malaria (clinical signs suggestive of malaria, but microscopy negative) and recorded as such together with the age of the patients: < 1 year, 1–4 years, 5–14 years, and 15+ years. For the present study, the aggregated monthly data, i.e. the numbers of confirmed and presumed malaria and the totals, were used.

### Birth weight data

All Rwandan PHCs record all births, birth outcomes and birth weights in specific birth registers. The birth registers from the 11 study PHCs were explored as the source for birth data in the present study. The information on births and outcome recorded during the first 15 days of every month from 2002 until 2007 were copied anonymized to case report forms (CRF). Collected data were: birth weight (in grams) for at-term, singleton deliveries, gestational age (in weeks of amenorrhoea), age of the mother, parity and the outcome of the pregnancy (at term delivery, premature birth, which is defined as <36 weeks of gestation as assessed by local standards, stillbirth or abortion). Individual data for malaria during pregnancy and body dimensions of the mothers were not available.

### Data handling and statistics

Data on CRFs from the birth registers were entered into a dedicated database written in SQL server 2005. All data were double entered and subsequently exported to MS Access (version 2003). The aggregated monthly malaria data were also imported in the same database. Incomplete records were excluded from analysis and obvious errors (age of mother older than 50 years, a term delivery of a baby with a birth weight lower than 1,500 g or higher than 6,000 g) in recorded data were also excluded.

For malaria incidence, the total incidence (the summation of the incidences of presumed and confirmed malaria) divided by the population size, was taken for the PHC at which the delivery took place and averaged over the nine months preceding the delivery. This incidence was linked to the individual birth data by PHC and month of delivery. The recorded premature delivery and stillbirth were recorded as obstetric events.

Birth weight was analyzed by using the actual numerical value in the statistical models. Low birth weight (LBW) was defined as a birth weight less than 2500 g. The association between malaria incidence and birth weight or obstetric events was first visually inspected by ranking the PHCs according to malaria incidence and plotting birth weight or the rate of obstetric events. Associations between birth weight and other factors and covariates were first explored with standard techniques such as regression and correlation analysis and analysis on variance (ANOVA).

The effect of malaria incidence on birth weight was investigated with mixed effects models with birth weight as the dependent variable, and malaria incidence as fixed effect. Other factors and covariates that showed a significant effect on birth weight were subsequently entered in the model. Bayes Information Criterion was used to compare models. The effect of malaria on the rate of obstetric events, stillbirth and abortion, was investigated with logistic regression models following a similar approach.

Statistical analysis was done with SPSS (v 15, SPSS inc. Illinois). Maps were plotted by Mapinfo (V 9, Mapinfo Inc, Golden USA).

## Results

### Description of data

Birth data were collected from 12,526 deliveries in the 11 different PHCs. Incorrect records of 280 deliveries were excluded, leaving 12,246 for analysis, comprising 11,879 at term deliveries, 149 premature deliveries and 218 still births. The data of eligible records are summarized in the Table 1. In all PHC registers birth weight as well as age of the mother was recorded. Data on parity and gravidity were not always present so that age was considered a proxy indicator of parity and gravidity. In analysing for birth weight, only normal and a term deliveries were used as the denominator. Births were not specified as to whether they were singletons or twins.

**Table 1:** General characteristics of studied communities

**Community**	**Province**	**Altitude (masl^1)^)**	**Population size**	**Annual malaria incidence^2)^**	**Classi-fication^3)^**	***Plasmodium *Prevalence (%)**	**No. studied deliveries**	**Maternal age in years, median, range)**	**Birth weight** **(g, mean, 95% CI)**	**Obstetric pathology^4)^**
Rukara	Eastern	1,612	43,943	20.4	Holo	>75	2,107	25 (15 – 50)	3214 (3195 – 3234)	1,5%

Bukora	Eastern	1,355	17,632	16.0	Holo	75	492	26 (17 – 45)	3239 (3198 – 3279)	3%

Busoro	Southern	1,478	24,146	19.2	Hyper	30 – 50	1,076	26 (16 – 48)	3048 (3022 – 3074)	15%

Mashesha	Western	1,239	32,015	25.4	Hyper	30 – 50	1,096	26 (16 – 48)	3181 (3155 – 3208)	10%

Karambi	Western	1,750	21,758	15.0	Meso	11 – 50	819	27 (17 – 48)	3024 (2993 – 3055)	0.6%

Mubuga	Western	1,656	13,232	17.0	Meso	11 – 50	400	26 (14 – 47)	3645 (3601 – 3690)	0.9%

Bungwe	Northern	2,393	26,690	14.5	Hypo	<3	1,033	26 (16 – 49)	3145 (3120 – 3170)	2%

Kivumu	Western	2.013	28,060	8.1	Hypo	<3	2065	23 (16 – 50)	3066 (3047 – 3085)	10%

Mbuga	Southern	2,528	39,153	2.4	Hypo	<3	692	24 (14 – 50)	3006 (2974 – 3037)	1.8%

Rwaza	Northern	1,749	18,808	15.1	Hypo	<3	663	26 (16 – 47)	3007 (2974 – 3040)	1.4%

Kicukiro	Kigali	1,567	33,010	-	Urban	NA	1,436	25 (14 – 45)	3139 (3116 – 3163)	0.2%

Legend: ^1) ^masl: meters above sea level; ^2) ^Mean annual malaria incidence/1000 capita: Total incidence: incidence of presumed malaria + incidence confirmed malaria; ^3)^The classical malariometric classification in holoendemic, hyperendemic, mesoendemic and hypo(epi-)endemic; "urban" indicates Kigali where there is no local malaria transmission; ^4)^the incidence of still birth plus the incidence of premature delivery.

### Effects on birth weight

**Figure 2:**
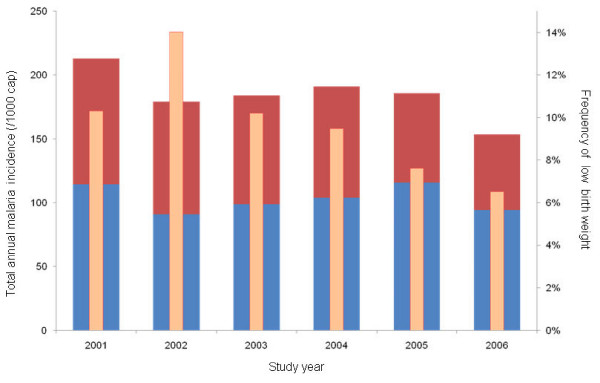
**Overall frequencies of low birth weight (narrow pink columns) among a term deliveries at the studied community health centers (right y-axis) and total annual incidence of presumed (wide blue columns) and confirmed (wide red columns) malaria per 1000 capita (left y-axis) in the same communities by years of study**.

There was a statistically significant difference in birth weights among health centers (Anova: p-value < 0.001) as well as in the frequency of the LWB (Chi square test: p-value < 0.001). However, this difference was mainly caused by the data from Mubuga. There was no explanation pointing at a structural error at this study site. Re-analysing the data without the data from Mubuga yielded similar results. Overall, there was a significant difference of birth weight between the years of study (Anova: p < 0.001). The lowest mean birth weight was recorded in 2003 after which it steadily increased. Figure 2 shows the annual incidence of the frequency of LBW. A significant seasonal effect on birth weight could be observed for all health centers (Anova: p-value < 0.001). Overall the highest mean birth weight was recorded between the months of June and September (dry season), and the lowest between the months of October and February. Altitude showed a slight but significant negative correlation with birth weight (Pearson correlation -0.088: p-value < 0.001). There was a small, but significant, positive association between birth weight and maternal age (Anova: p-value < 0.001).

**Figure 3:**
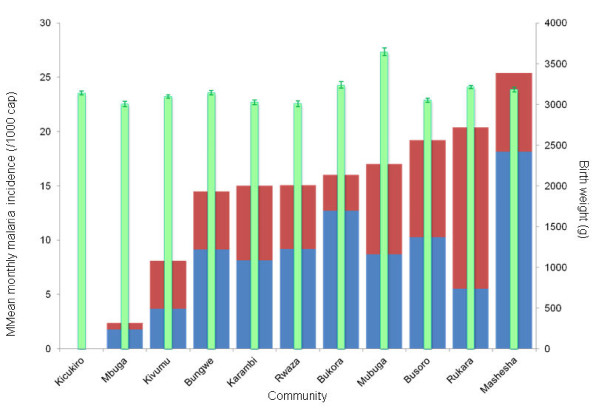
**Mean birth weight (g) of each of the 11 participating primary health centres recorded during the whole study period (green columns, right y-axis)) and mean monthly incidence of presumed (blue columns) and confirmed (red columns) malaria per 1000 capita for the health centres (left axis), ranked by total incidence (means over the years of study)**. The errors bars indicate the 95% confidence interval of the mean birth weight.

The mean birth weight for each health center and the mean monthly incidence of confirmed and presumed malaria (per health centre) is presented in Figure 3, which clearly shows the lack of any association between birth weight and malaria incidence by PHC. The final mixed effects model included birth weight as dependent variable, malaria incidence, age of the mother, month of the year and study year as fixed covariates or factors, respectively. Entering PHC as an additional factor did not improve the model. Entering altitude as a covariate showed a slightly negative effect of altitude on birth weight (p-value < 0.001) but since this did not improve the model, altitude was not included in the final model. The final model showed a small but not significant effect of malaria incidence on birth, i.e. the higher the malaria incidence, the lower the birth weight (p-value < 0.098). Maternal age and year of study still showed a positive effect on birth weight.

### Obstetric events

**Figure 4:**
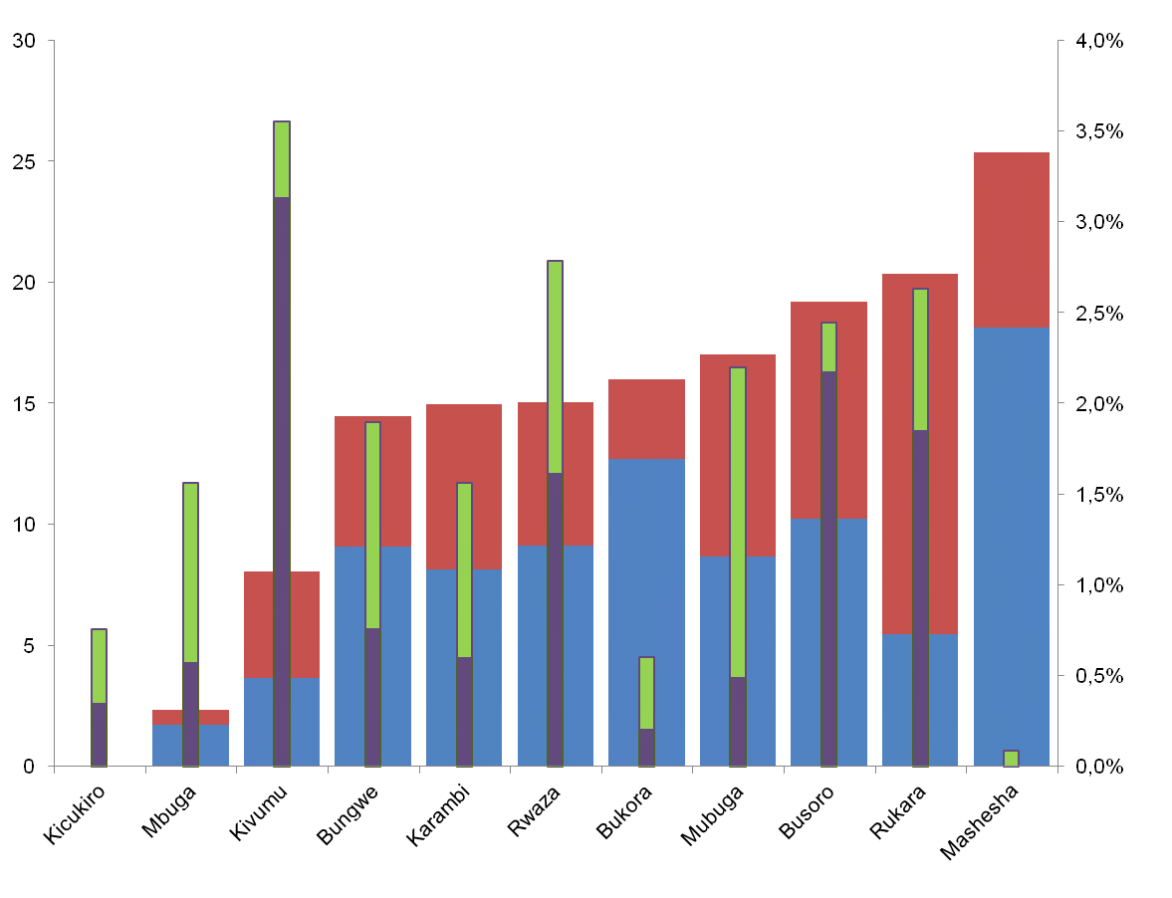
**Frequencies of obstetric events: still births (purple columns) and premature deliveries (green columns) recorded during the whole study period (right y-axis) and mean monthly incidence of presumed (blue columns) and confirmed (red columns) malaria per 1000 capita (left y-axis), ranked by total incidence (means over the years of study)**.

Malaria incidence and obstetric events, i.e. pre-mature delivery, and still births, by PHC, are presented in Figure 4. The final logistic regression model included PHC, month of the year, study year and maternal age and as fixed factors or covariate, respectively. There was a slight but significant decreasing effect of malaria incidence on the occurrence of premature delivery (OR: 4.5•710^-10^, 95% CI: 3.32•10^-19 ^– 0.628; p-value 0.045) and stillbirth (OR: 4.2•10^-11^, 95% CI: 6.41•10^-19 ^– 0.003; p-value 0.009). There was also a slight trend towards declining frequencies of stillbirth and pre-mature delivery over the years (overall p-value < 0.001).

## Discussion

This study confirms that in areas with low to moderate malaria transmission intensity, such as in Rwanda, the complications of malaria in pregnancy are not dominated by low birth weight. Acute symptomatic malaria infections that may cause acute obstetric complications were not reflected in a higher number of premature deliveries and stillbirths in this study.

The present research is the first study performed retrospectively on the outcome of pregnancy in the face of a changing pattern of malaria incidence. The strength of the current study is the large sample size that covers a wide geographical range and time interval. The use of stored information also has its limitations. For example, it cannot be explained why the birth weight data from Mubuga are on average higher compared to other PHCs. It cannot be assessed whether a systematic error in recording these data has been made or that indeed the general birth weight is higher in this part of the country due to other, for example nutritional, factors. Data of malaria in the individual pregnant women were not available and the risk of malaria that was used in this study applies to the general population, which can be different from the risk of pregnant women. The pregnant women who deliver at the PHC probably reflect the same population as patients who seek help for symptomatic malaria, but this could not be confirmed in this study.

Malaria (in pregnancy) was considered a major health problem in Rwanda [14] but recently the incidence of the symptomatic disease and malaria related mortalities, significantly decreased [13]. This was attributed to an effective combination of mass distribution of long-lasting insecticide-treated bed nets (LLIBN) and artemisinin-based combination therapy (ACT). Nowadays, malaria transmission intensity is considered to be low to moderate and thus chronic or repeated malaria in pregnant women should occur less frequently resulting in a reduction of the incidence of LBW. This is only partly confirmed in the present study as there was no association between birth weight and malaria incidence by PHC, but a general increase in birth weights over the study years was observed.

The decline of malaria incidence over the years as reported by National Malaria Control Programme of Rwanda (unpublished data) coincided with the secular trend in birth weight but was not the cause. This shows that, independent from malaria, the conditions of pregnant women are steadily improving. Improved socio-economic factors and consequent improved nutritional status probably play a significant role. In addition, the implementations of vigorous malaria control methods have contributed to the reduction of the disease in Rwanda and this clearly has positive effects on general well-being.

The implication of the findings in the present study is that in low to moderate transmission regions such as Rwanda, malaria does not cause LBW. It is mainly a problem of the high transmission regions. The pathophysiology of malaria during pregnancy in holoendemic regions is characterized by chronic or repeated placental infection, inflammation and eventually dysfunction that leads to LBW. In low transmission areas, with low levels of immunity, pregnant women probably have an equal chance as non-pregnant individuals for developing symptoms that will lead to treatment. The disease is thus dominated by a short episode of very symptomatic acute malaria. This has different effects on placental function and fetal conditions than chronic placental infections.

The interaction between birth weight and age of the mother (weight increases with mothers' age), which was also found in Tanzania where it was reported that the risk of delivering a baby with LBW was two fold higher in women below 20 years compared to women in age group 20 – 34 years [16].

There was a seasonal relationship in birth weight with an increase in the period June – September, the dry season, of each year. A similar seasonal effect was also found in Tanzania [16]. This period coincides with the period of rest after the harvesting season and could explain the seasonal increase in birth weight. Food is abundant in this season and women are often not working the field at this time. Rest during the second and third trimester increases the birth weight and in combination with food this is probably an important determinant for higher birth weight.

The present study also reports a negative correlation with birth weight and altitude. In Tanzania, Mmbando and co-workers found a significant decreasing trend of the incidence of LBW from rural lowlands to highlands [16].

There was a slight trend towards declining frequencies of stillbirth and pre-mature delivery over the years. A striking observation is that in Mashesha the highest incidence of malaria was found, but only few cases of premature delivery and no stillbirths occurred. In contrast, the lowest malaria incidence outside Kicukiro (Kigali), was recorded in Mbuga, where a very average number of stillbirths and pre-mature deliveries were observed.

Interestingly, Rwanda had and is still not having specific interventions for pregnant women but takes preventive measures aiming as the population as a whole. Although malaria does not seem to have a major effect on birth weight in Rwanda, pregnant women and their unborn babies are still a very vulnerable group and are at risk for severe effects of malaria episodes during pregnancy. Therefore special attention should be given to this group, also in the absence of low birth weight. In addition trends or fluctuations of malaria in pregnancy should continuously be monitored so that, if needed, specific measures can be taken such as the reintroduction of Intermittent preventive therapy in pregnancy, targeted education for this specific group about risks and preventive measures or targeted bed net distribution.

## Conclusion

Malaria is not the main determinant of birth weight in Rwanda. Further increase in birth weight could be achieved by nutritional improvements and socio-economic development. Malaria specific interventions remain important and special attention should be given to pregnant women, although they may not increase mean birth weight.

## Competing interests

The authors declare that they have no competing interests.

## Authors' contributions

SR: design and coordination of the study and supervision of data collection and entry, data analysis, drafting of manuscript. PFM: design of the study, interpretation of data, critical reading and modifications of the manuscript, principle investigator. CK: making PNLP malaria data accessible. HDFHS: conception and design of the study, critical reading and modifications of the manuscript. NK: data collection. JV: day-to-day overall supervision of the study. PJdV: conception and design of the study, analysis and interpretation of data, critical reading and modifications of the manuscript, principle investigator
